# Urinary microvesicles: a window into the kidney

**DOI:** 10.1093/ckj/sfaf189

**Published:** 2025-06-17

**Authors:** Luisa Schnobrich, Hayo Castrop

**Affiliations:** Institute of Physiology, Faculty of Biology and Preclinical Medicine, University of Regensburg, Regensburg, Germany; Institute of Physiology, Faculty of Biology and Preclinical Medicine, University of Regensburg, Regensburg, Germany

**Keywords:** biomarkers, diagnosis, kidney disease, prognosis, urinary extracellular vesicles

## Abstract

Chronic kidney disease (CKD) is a growing concern in aging populations. CKD is characterized by two hallmark symptoms: a decline in the glomerular filtration rate (GFR) and albuminuria. Early changes in kidney function are notoriously underdiagnosed, suggesting the need for new noninvasive diagnostic and prognostic biomarkers of CKD. Thus, analysis of urinary extracellular vesicles (uEVs) may broaden the diagnostic options for CKD. EVs are a heterogeneous group of particles, enclosed by a lipid bilayer, which differ in size, biogenesis, and function. EVs can be readily recovered from the urine (urinary EVs, uEVs), where they are derived from various cells of the kidney, bladder, prostate, and utero-vaginal tract. Within the kidney, EVs are released by almost all cell types, including but not limited to podocytes, cells of the proximal and distal tubules, the collecting duct, and the loop of Henle. In addition to specific markers of parental cells, uEVs carry mRNAs, miRNAs, and proteins. Thus, analysis of uEVs may provide insights into the content and composition of the specific cells from which they are released, leading to the identification of new diagnostic and prognostic biomarkers for kidney diseases of different etiologies. This review provides an overview of kidney disease-related changes in uEV size and concentration and covers the potential of uEVs as new biomarkers for various types of kidney disease.

## INTRODUCTION

Chronic kidney disease (CKD) is a growing concern in aging populations [1]. Independent of its etiology, CKD is characterized by two hallmark symptoms: the first is a decrease in the glomerular filtration rate (GFR), and the second is an increase in the excretion of urinary proteins, including albumin (proteinuria/albuminuria) [2]. The decline in the GFR in most cases is progressive and follows variable kinetics [3]. Albuminuria is the consequence of compromised integrity of the glomerular filtration barrier and/or inadequate, saturated tubular protein reabsorption [4]. In the early stages of compromised kidney function, both symptoms frequently remain undetected. Thus, the estimation of the GFR by routinely used methods, such as the measurement of creatinine and cystatin C, has considerable uncertain effects for patients with minor/early reductions in the GFR [5]. Similarly, the increased glomerular filtration of albumin, caused by the compromised integrity of the glomerular filtration barrier, is masked by increased proximal tubular reuptake for prolonged periods of time and becomes apparent only when the tubular reuptake is saturated or if the tubular function itself is compromised, bearing again the risk of underdiagnosis of early functional changes [4]. Consequently, renoprotective measures are often initiated late, when CKD has already advanced.

In the search for new biomarkers of early CKD, renal extracellular vesicles (EVs), which are readily recovered from the urine (uEVs), may serve as noninvasive diagnostic and prognostic carriers of information regarding the function and integrity of the glomerular filtration barrier and the tubular system [6].

The biological functions of EVs include the maintenance of cellular homeostasis, and the establishment of intercellular communication pathways [7]. For example, EVs have been shown to mediate the transfer of signals between the proximal and distal portions of the nephron. Furthermore, uEVs are involved in the communication between the glomerulus and the tubular system [7, 8], the intraglomerular signaling, and the signaling between tubular and interstitial cells [7]. EVs interact with target cells by three different mechanisms: first, the activation of receptors of the target cell; second, the membrane fusion with the target cell; and, finally, the uptake of EVs into the target cells via endocytosis. The last two mechanisms lead to an incorporation of EV cargo into the target cells [7]. The uptake of uEVs by recipient cells is a regulated process. Thus, the uptake of uEVs by collecting duct cells is regulated by vasopressin *in vivo* and *in vitro* [9, 10]. In addition, uEVs may regulate the transport of water and ions and control the intrarenal renin-angiotensin system [8]. Furthermore, stem cell-derived EVs may contribute to renal repair [11]. Under pathophysiological conditions, the formation of EVs and the signaling pathways mediated by uEVs both may exacerbate tubular and glomerular injuries [7, 8, 11].

EVs in the urine are released from various cells of the kidney, bladder, prostate, and utero-vaginal tract, including the uterus and vagina, as well as from residing bacteria [6]. Within the kidney, EVs are derived from various structures, including but not limited to podocytes, cells of the proximal and distal tubules, and the collecting duct [6].

Despite their marked heterogeneity, EVs share the common feature of containing cytoplasm and membrane constituents of the cell of origin [12], thus providing insights into the content and composition of the specific cells from which they are released.

Cellular abnormalities, which may be identified by assessment of the content of urinary EVs, include markers of inflammation, profibrotic factors [13], and markers of apoptosis [14].

In this review, we will (i) provide an overview of the mechanisms of renal extracellular vesicle formation, (ii) report the sources of urinary EVs, (iii) address the concentration and content of extracellular renal vesicles in health and disease, and (iv) discuss the potential of specific vesicles as new, noninvasive biomarkers of the structural and functional status of the kidney.

## BIOGENESIS OF EXTRACELLULAR VESICLES

EVs are released from cells and are enclosed by a lipid bilayer [15].

Based on their size, biogenesis and function, three distinct types of EVs are distinguished: namely, exosomes, microvesicles (also known as ectosomes), and apoptotic bodies [16, 17]. In addition, various other subtypes of EVs have been classified recently, including but not limited to migrasomes, which are formed by migrating cells [18, 19]; large oncosomes, which are derived from the plasma membrane of tumor cells; and exophers, which eliminate damaged organelles and proteins from healthy cells [18].

**Figure 1:**
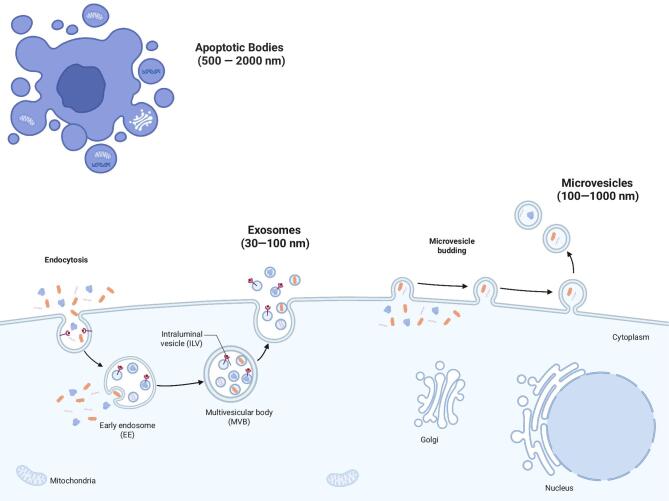
Simplified depiction of the biogenesis processes of exosomes, microvesicles, and apoptotic bodies. As part of the cellular endosomal pathway ILVs are formed within early endosomes, thus generating MVBs. The fusion of the latter with the plasma membrane leads to the release of ILVs into the extracellular space in form of exosomes. Microvesicles are generated by direct outward budding and pinching off the plasma membrane, whereas apoptotic bodies are released during apoptosis.

Exosomes, ranging from 30 to 150 nm in size, are formed during the maturation of multivesicular bodies (MVBs), which are part of the cellular endosomal pathway. As part of this process, intraluminal vesicles (ILVs) are formed within early endosomes, thus generating MVBs. The fusion of the latter with the plasma membrane leads to the release of ILVs into the extracellular space (Fig. 1). The formation of ILVs and the sorting of specific cargo are facilitated by endosomal sorting complex required for transport (ESCRT)-dependent and ESCRT-independent mechanisms. ESCRT-independent pathways rely on neutral sphingomyelinase 2 (nSMase2), as well as components of lipid rafts and tetraspanin-enriched domains. A more in-depth description of the different pathways of ILV formation can be found in [20].

Microvesicles, also known as ectosomes [21, 22], range from 100 to 1000 nm in size [16] and are generated by the direct outward budding and pinching of the plasma membrane (Fig. 1) [21, 22]. In this pathway, specific Ca^2+^-dependent proteins (e.g. flippases, floppases, scramblases, and calpains) lead to membrane asymmetry [23, 24], whereas changes in the lipid and protein composition of the plasma membrane cause alterations in the membrane curvature and rigidness [22], eventually leading to membrane blebbing/outward budding [22, 23]. The shedding and fission of microvesicles occur via ATP-dependent contraction, which is mediated by the interaction of actin and myosin filaments [22]. Although the formation of exosomes and microvesicles constitutes two distinct biological processes, proteins shared between both pathways have been described [22, 23]. For example, TSG101 is involved in the ESCRT-dependent pathway of exosome formation and the release of microvesicles [22].

Apoptotic bodies, the largest subtype of EVs (500–2000 nm), are released during apoptosis (Fig. 1) [16]. The functional properties and mechanistic details of the formation of apoptotic bodies are not fully understood. However, increasing evidence suggests that apoptotic bodies transport various contents. Apoptotic bodies represent a subgroup of apoptotic EVs that are particularly large [25].

The International Society for Extracellular Vesicles recommends the use of the generic term EVs instead of specific but sometimes poorly defined terms related to biogenesis pathways. Operational terms, such as size, density, and biochemical composition, may be added as prefixes to the term EV [15].

## ISOLATION OF (urinary) EVs

Different methods have been developed to isolate EVs from body fluids, including the urine. The most commonly used techniques to isolate EVs are ultracentrifugation and precipitation-based techniques [26, 27]. Ultracentrifugation is commonly used in density gradient media. This method allows the separation of vesicles from other urinary constituents based on their density [27]. Furthermore, based on their surface characteristics, EVs can be precipitated employing commercial kits and polymers such as polyethylene glycol [27, 28].

**Figure 2:**
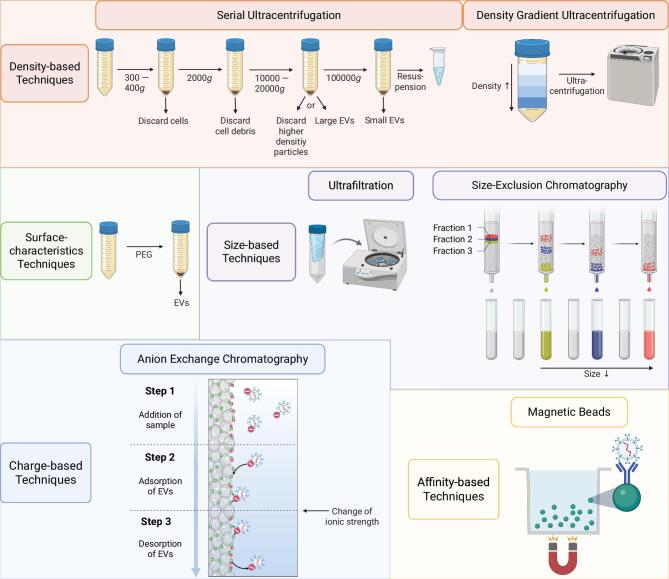
Summary of the commonly used methods to isolate EVs from various sample matrices. EVs are isolated based on their surface characteristics, density, size, and charge [26, 27, 97, 98]. PEG, polyethylene glycol.

Modern isolation and separation techniques for EVs can be distinguished in size-, charge-, and affinity-based approaches. Size-based techniques include size-exclusion chromatography (SEC), different filtration approaches, and flow field-flow fractionation. Charge-based separation and isolation methods of EVs use ion-exchange techniques, electrophoresis, and dielectrophoresis. Affinity chromatography and immunocapture, as affinity-based isolation techniques of EVs, allow a selective isolation of EVs based on their origin from different cell types [26]. A more in-depth description of the aforementioned methods to isolate EVs, including their advantages and pitfalls can be found in [26]. The commonly used methods of EVs isolation are summarized in Fig. 2.

The existing isolation methods, based on UC and precipitation, are often compromised by low yield, purity, and the lack of consistency [29]. Due to the heterogeneity of EVs, a combination of methods is often the superior approach to isolate EVs of sufficient yield and specificity [26]. Thus, a modified method was developed employing a combination of ultrafiltration, ultracentrifugation and SEC. This method facilitates the highly reproducible isolation of EVs from urine within a size range of 50–150 nm [29].

A common challenge when isolating uEVs is the presence of large amounts of Tamm–Horsfall protein (THP) in the urine. Under physiological conditions, THP is the most abundant protein in the urine, forming high-molecular-weight polymeric networks, which have been shown to entrap EVs, thus reducing the reproducibility of the isolation procedure [30]. Furthermore, EVs entrapped in THP networks can be lost during low speed centrifugation steps, and THP linked to EVs may sediment with the EVs during ultracentrifugation, thus interfering with proteomic studies [31]. Different methods have been described to remove THP including, but not limited to, the treatment with dithiothreitol (DTT) [30], 3-[(3-cholamidopropyl)dimethylammonio]-1-propanesulfonate [32], salt precipitation [33], hydrostatic filtration dialysis, [34] addition of various chaotropic reagents [35], and dilution under alkaline conditions [31].

The isolation of EVs from nephrotic urine is particularly challenging. Thus, highly abundant soluble proteins reduce the ultrafiltration efficacy by obstructing the nanomembranes. These proteins, when attached to uEVs, are recovered after ultracentrifugation, hampering the detection of less abundant microvesicular proteins by mass spectrometry. Nevertheless, by combining ultracentrifugation and SEC, the isolation of EVs from nephrotic urine was improved [36].

## ORIGIN AND SOURCES OF URINARY EVs

EVs are secreted by nearly all cell types into the extracellular space. Accordingly, nearly all cell types in the kidney have been shown to produce/secrete EVs *in vitro* and *in vivo*.

Using an immortalized podocyte cell line, Burger *et al.* showed that different stressful stimuli (for example, elevated glucose concentrations or mechanical stress) lead to increased production of podocyte-derived microparticles [37]. Stimulation with high glucose levels also led to the release of an increased number of exosomes in cultured human mesangial cells [38] and mouse primary kidney glomerular endothelial cells (GECs) [39–41]. EVs have also been isolated from conditionally immortalized human podocytes, mesangial cells, glomerular endothelial cells, and proximal tubular cells [42]. Furthermore, EVs were isolated from RG1 cells, a murine juxtaglomerular cell line [10], and primary mouse parietal epithelial cells (PECs) [43]. Recent findings suggest that macula densa cells may also produce and secrete EVs. Thus, major cell processes, which are primary and thick cytoplasmic protrusions and hair-like processes projecting from either the cell body or major processes, have been identified at the base of macula densa cells. Inside the hair-like processes, cytoplasmic vesicles moving between the bodies of macula densa cells and the tips of the processes were observed, indicating that the minor processes play a role in the endocytosis and secretory functions of macula densa cells [44].

**Figure 3:**
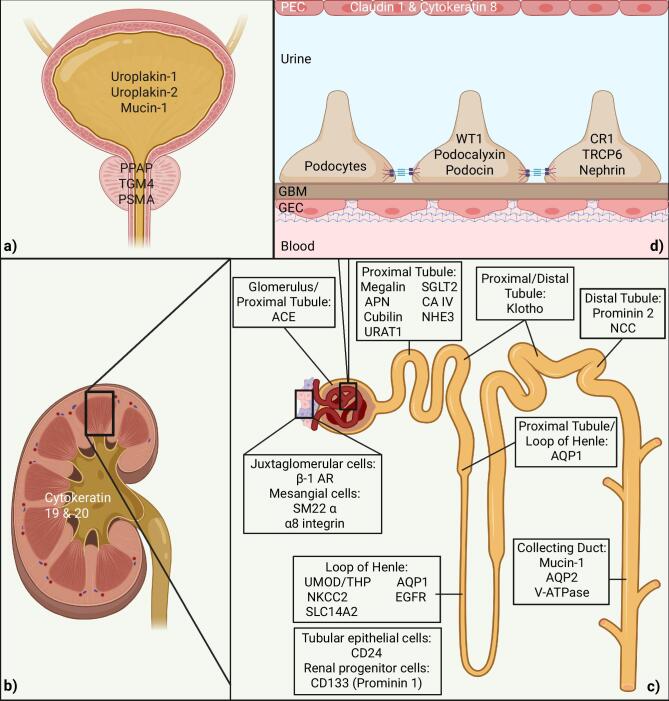
Depiction of uEV markers for different cell types of the urinary tract. Depicted are markers for bladder and prostate (**a**), renal pelvis (**b**), tubule system and juxtaglomerular apparatus (**c**) and the glomerulus (**d**). PPAP, prostatic acid phosphatase; TGM4, prostate transglutaminase; PSMA, prostate-specific membrane antigen; ACE, angiotensin-converting enzyme; APN, aminopeptidase N; URAT1, urate transporter 1; SGLT2, sodium/glucose cotransporter 2; CA IV, carbonic anhydrase, NHE3, Na^+^/H^+^ exchanger isoform 3; NCC, thiazide‐sensitive Na‐Cl cotransporter; AQP2, aquaporin 2; V-ATPase, vacuolar-type H^+^-ATPase; AQP1, aquaporin-1; UMOD/THP, uromodulin/Tamm–Horsfall protein; NKCC2, Na‐K‐2Cl cotransporter; SLC14A2, Solute Carrier Family 14 Member 2; EGFR, epidermal growth factor receptor; β-1 AR, beta-adrenergic receptor-1; SM22-α, smooth muscle 22 alpha; α8 integrin, alpha-8 integrin; WT-1, Wilms’ tumor 1; CR1, complement receptor 1; TRCP6, canonical transient receptor potential 6.

Additionally, EVs from most cell types of the kidney have been isolated from human urine. Different markers have been used to map urinary EVs to their cells of origin. Thus, markers of podocytes, proximal tubular cells, renal progenitor cells, cells of the loop of Henle, distal convoluted tubules, and collecting ducts have been described [6]. Moreover, specific populations of urinary EVs from cells of the glomerulus and the nephron were quantified. To assess the origin of EVs within given structures of the kidney more specifically, markers for the identification of uEVs originating from juxtaglomerular cells (β-1 AR), mesangial cells (alpha-SM22), parietal cells of Bowman's capsule (claudin 1, cytokeratin 8), and the transitional epithelium of the renal pelvis (cytokeratins 19 and 20) were used [45]. In addition to urinary EVs, mesangial cell-derived EVs have been found in plasma samples, as identified by the presence of α8-integrin [46]. A summary of the markers of the origin of EVs is shown in Fig. 3.

The generation and release of EVs from these cell types occur under normal conditions [10, 19, 38–43, 47] and may be altered in diseased kidneys.

## uEVs In Health And Disease: Size And Concentration In The Urine

The content of EVs has been extensively studied (see [48] and [49]). Currently, Vesiclepedia, a community compendium for EVs and particles, comprises 3481 studies and contains almost 567 000 entries for proteins; >20 000 entries each for miRNAs and mRNAs; and entries for lipids, DNA, and metabolites from 56 different species [49]. Out of 3481 studies, 114 addressed EVs from human urine samples [50]. Approximately 25% of all human proteins are secreted via exosomes [51].

Over the last few years, changes in EV size and concentration and alterations in EV content have been investigated as potential new biomarkers of different kidney diseases. The most promising results are addressed in the following chapter.

### Concentration and size of urinary EVs as biomarkers of kidney function

The total uEV concentration and the concentration of a distinct uEV size range (0.1–0.5 µm) were significantly greater in patients with lupus nephritis (LN) than in patients with SLE without kidney involvement. Furthermore, the concentration of the uEV size subsets ranging from 0.3–0.5 µm differed markedly among patients with SLE with (LN) and without kidney involvement [52]. In contrast, among patients with Dent's disease type 1, the relative concentration of uEVs in the urine was lower than that in healthy controls, which may simply be related to the polyuria observed in patients with Dent's disease. In addition, the uEVs of control participants were significantly smaller than the uEVs of patients with Dent's disease [53]. The size differences observed between healthy controls and patients with Dent's disease were attributed to mutations in CLCN5, which led to alterations in ClC-5 localized in early endosomes, thus impacting the production and release of exosomes [53]. Consistent with the results for patients with Dent's disease, the mean size of uEVs was lower in healthy controls than in patients with focal segmental glomerulosclerosis (FSGS) [54]. The total uEV concentration was also greater in patients requiring hemodialysis following COVID-19 infection than in other critical patients with COVID-19. Furthermore, hemodialysis as a clinical outcome in critical patients with COVID-19 was predicted by the total uEV concentration [55]. The uEV concentration also differed between different groups of children with idiopathic nephrotic syndrome (INS) and healthy controls. Furthermore, the size of uEVs was significantly different between individuals with different INS disease groups [56].

Finally, studies in different organ or cell contexts indicate that inflammation alters the biogenesis and release of EVs [52]. For example, changes in EV size distribution and concentration in microglia following LPS activation have been described [57]. Thus, it seems plausible that inflammation in the kidney/urogenital tract also leads to changes in the size and concentration of uEVs.

In summary, the size and concentration of uEVs might be promising biomarkers for different kidney diseases or kidney-related outcomes, possibly allowing for conclusions to be drawn about the state of the kidney.

In addition to changes in the total uEV concentration, differences in the concentrations of specific subtypes of uEVs have been observed between healthy participants and patients with renal disease [19, 58, 59]. Specifically, the median concentrations of total large urinary EVs and the large podocyte-specific EVs were significantly greater among children with INS who had relapsed than among those in remission [58]. Similarly, the TSPAN4-migrasome (500–3000 nm) concentration was increased in a cohort of patients with varying kidney diseases diagnosed by renal puncture biopsy, irrespective of eGFR or 24-h proteinuria, compared with healthy controls. Similarly, the TSPAN4-migrasome signal was elevated in patients with kidney disease with normal eGFRs (>90 ml/min/1.73 m^2^) and 24-h urine protein levels (<150 mg) compared with healthy controls. Furthermore, the TSPAN4-migrasome signal was increased in patients with diabetic nephropathy, membranous nephropathy, focal segmental glomerulosclerosis, and other kidney diseases compared with healthy controls. The TSPAN4-migrasome signal was also shown to be a marker for distinguishing patients with kidney disease from healthy controls via receiver operating characteristic curve analysis. However, the TSPAN4-migrasome signal did not allow for differentiation among patients with different kidney diseases. Furthermore, the percentage of podocyte-derived migrasomes was significantly greater in patients with kidney disease than in controls [19]. Miller *et al.* reported increased urinary EV concentrations in patients with acute kidney injury (AKI) post-cardiac surgery compared with patients who did not develop AKI. Before cardiac surgery, the urinary concentration of podocalyxin-positive exosomes was significantly lower in patients who developed AKI after surgery than in patients with preserved kidney function. Consequently, a model including preoperative podocalyxin-positive exosomes showed good predictive value for AKI post-cardiac surgery [59].

In contrast to previously described results, no difference in uEV concentration was observed between patients with focal segmental glomerulosclerosis and healthy controls [54]. uEV size and concentration also did not differ between patients with IgAN and healthy controls [60]. These data suggest that the concentration and size of uEVs do not generally change in patients with kidney disease and, consequently, may be used as biomarkers for specific kidney diseases and as predictors of the course of kidney disease. Consistent with this assumption, the urinary TSPAN4-migrasome concentration progressively increased from CKD stages 1–3 and subsequently decreased in the latter stages. This increase in the TSPAN4-migrasome signal may be related to the deterioration of podocyte integrity, whereas the decrease in the signal in the advanced stages of kidney disease may mirror the progressive loss of podocytes, given that most urinary migrasomes are of podocyte origin [19].

In summary, changes in the concentration and size of specific uEV subgroups, which are dependent on the cellular origin, may lead to the identification of new biomarkers for different kidney diseases.

### Content of specific uEVs

#### RNAs in urinary EVs as biomarkers of kidney disease

mRNAs, miRNAs, circRNAs, lncRNAs, and tsRNAs in uEVs have been investigated as biomarkers in attempts to diagnose kidney diseases, pinpoint different etiologies, and predict the future progression of kidney diseases.

High-throughput sequencing techniques revealed that multiple RNAs were differentially expressed in uEVs between healthy participants and/or other disease groups and patients suffering from IgA vasculitis (IgAV) [61], FSGS [62], IgA nephropathy [63, 64], type II diabetes [65], diabetic nephropathy [62, 66], ADPKD [67], diabetic kidney disease (DKD) in type I diabetes [68], Fabry nephropathy [69], systemic lupus erythematosus [70], CKD [71], AKI [59], idiopathic membranous nephropathy (IMN) [72], and AAV [73]. From the broad array of differentially expressed vesicular RNAs, several candidates were specifically assessed that may serve as biomarkers for diagnostic and prognostic purposes, as outlined in the following chapters.

### miRNAs

#### Glomerular diseases

Glomerular diseases are a diverse group of kidney disorders of various etiologies, including but not limited to FSGS, IgAN, and IMN. They are characterized by inflammation and immune-mediated processes [72, 74] or by podocyte damage and loss [75]. Several miRNAs may serve as potential biomarkers of glomerular diseases. Thus, miR-30b-5p and miR-9–5p, which were significantly more abundant in the uEVs of healthy controls than in those of patients with IMN, have been proven to be useful biomarkers for IMN [72]. Similarly, miR-451a and miR-7d-3p, which were both upregulated in the uEVs of patients with IgAN compared with those of healthy controls, both alone and in combination, have high diagnostic value for IgAN [64]. Furthermore, the expression of miR-21, miR-30a, miR-196a, and miR-200a was increased in the uEVs of patients with FSGS compared with healthy controls [62].

#### Metabolic nephropathies/diabetic kidney disease

uEV-miRNAs have been extensively studied as biomarkers of diabetic nephropathy. Significant changes in the expression of vesicular miR-151a-5p, miR-182–5p, miR-1307, miR-320a, and miR-423–5p were detected between patients with T2D with and without diabetic nephropathy. Receiver operating characteristic curve analysis revealed the potential of these miRNAs as biomarkers for the early detection and monitoring of the development of diabetic nephropathy in patients with type 2 diabetes [65]. Furthermore, Ali *et al.* reported 13 miRNAs in uEVs, including miR-30b-5p and miR-29c-3p, as differentially expressed between patients with type 2 diabetes and healthy controls [65]. Additionally, the expression of miR-21 was found to be lower in the uEVs of patients with diabetic nephropathy than in those of healthy controls [62]. In addition to 11 other miRNAs, miR-320b and the previously mentioned miR-30b-5p were also identified to be common and differentially expressed in EVs isolated from plasma and urine samples of patients with T2D and DKD compared with those from controls [66]. Several authors have reported that the concentrations of different miRNAs in uEVs were dependent on albuminuria [65, 76]. Thus, the expression levels of vesicular miR-151a-5p, miR-182–5p, miR-1307, miR-320a, and miR-423–5p were significantly higher in patients with T2D with and without diabetic nephropathy with macroalbuminuria than in normo- or microalbuminuric patients [65]. Furthermore, both miR-126 and miR-145 were significantly more highly expressed in the uEVs of proteinuric patients with T2D than in those of normoalbuminuric patients with T2D, regardless of their micro- or macroalbuminuria status [76].

#### Acute kidney injury

The sequencing of RNAs isolated from uEVs revealed six differentially expressed miRNAs: among patients who developed AKI post-cardiac surgery, the expression of three miRNAs was lower before and that of three other miRNAs was lower after cardiac surgery compared with levels in patients who did not develop AKI. uEV-miR-125a-5p in combination with other variables was shown to be a good predictor of AKI [59].

#### Vasculitides

Vasculitides are a group of diseases characterized by inflammation of blood vessel walls [77]. In this context, changes in uEV-miRNA expression have been investigated in patients suffering from antineutrophil cytoplasmic antibody (ANCA)-associated vasculitis (AAV) and IgAV.

miR-30a-5p and miR-182–5p, among other miRNAs, were found to be significantly increased in the uEVs of patients with AAV compared with those of healthy controls. Bioinformatic analysis of the targets of the five upregulated miRNAs in patients with AAV provided insights into the role of the identified miRNAs in AAV pathogenesis [73]. High-throughput sequencing identified 57 differentially expressed miRNAs in uEVs between patients with IgA vasculitis with and without nephritis, among which five miRNAs (hsa-miR‐3065‐5p, hsa-miR‐383‐5p, hsa-miR‐25‐3p, hsa-miR‐450b‐5p, and hsa‐miR‐499a‐5p) showed diagnostic value in IgAVN, with hsa-miR-383–5p being the most promising biomarker candidate [61].

#### Genetic nephropathies

uEV-miRNAs have also emerged as potential biomarkers for genetic nephropathies, including but not limited to ADPKD and Fabry disease/nephropathy. Fabry nephropathy is caused by a mutation in the *GLA* gene leading to the accumulation of globotriaosylceramide and its deacetylated form, globotriaosylsphingosine, in all renal cells [69], whereas ADPKD is characterized by the formation of fluid-filled cysts in the kidney caused by mutations in the *PKD1* or *PKD2* gene [67]. Compared with those in healthy controls, miR-222–3p and miR-21–5p levels were significantly upregulated in the uEVs of patients with Fabry nephropathy with stable renal function. Additionally, vesicular miR-222–3p and miR-21–5p levels were upregulated in patients with progressive Fabry nephropathy compared with healthy controls, whereas uEV-miR-30a-5p, uEV-miR-204–5p, and uEV-miR-10b-5p were significantly downregulated when these two groups were compared. Significant differences in the expression of vesicular miR-30a-5p, miR-204–5p, and miR-10b-5p were observed between the two patient groups. All five differentially expressed miRNAs, either alone or in different combinations, showed high discriminatory power to distinguish between healthy controls and patients with progressive Fabry nephropathy [69]. Similarly, vesicular miR-29c-3p, miR-320b, miR-320c, and miR-1246 might be used to differentiate between patients with ADPKD and healthy controls [67].

### tsRNAs

tsRNAs, which are derived from mature tRNA molecules, can be distinguished into tiRNAs and tRFs (tRNA-derived fragments). Even though they are a class of noncoding RNAs, tsRNAs have a multitude of regulatory functions and are thus significantly involved in cell function and the development of different diseases [78]. tRF3-Ile-AAT-1 and tiRNA5-Lys-CTT-1, which are increased in the uEVs of patients with LN compared with those of patients with SLE without LN and healthy controls, have shown potential for diagnosing and predicting nephritis in patients with SLE [70].

### circRNAs

CircRNAs are characterized by their covalently closed structure, which prevents degradation by exonucleases, thus giving them high biostability in comparison to linear RNAs. CircRNAs play diverse roles in gene transcription, protein translation and disease processes [79]. Cao *et al.* investigated the potential of exosome-derived circRNAs as biomarkers for chronic renal fibrosis. Both hsa_circ_0036649 and hsa_circ_0008925 could effectively distinguish renal fibrosis in different patient groups [71, 80]. Furthermore, 123 circRNAs were found to be differentially expressed in uEVs between patients with type II diabetes with and without diabetic nephropathy [81].

### lncRNAs

Long noncoding RNAs are RNA molecules longer than 200 nucleotides with crucial roles in the regulation of gene expression and other cellular processes [82]. lncRNAs in uEVs have shown potential as biomarkers for prostate, bladder, and renal cell carcinomas [83]. Furthermore, 126 uEV-lncRNAs were found to be dysregulated between patients with type II diabetes with and without diabetic nephropathy [81], suggesting their potential as biomarkers for kidney diseases.

### mRNAs

The expression level of a uEV mRNA-based stress score, consisting of six genes (*GPX3, NOX4, MSRB1, MSRA, HRSP12*, and *CRYAB*), was found to be higher in patients with diabetes (T1D and T2D) with albuminuria than in normoalbuminuric individuals. Furthermore, the stress score reflected long-term deterioration of kidney function. Most notably, the stress score was capable of detecting early stages of kidney function decline in normoalbuminuric individuals. Thus, the stress score has the potential to identify patients with declining kidney function and a greater risk of developing DKD [68]. In addition to their potential as biomarkers for different diseases, uEV-mRNAs show promise as biomarkers for the efficacy of different drugs in the treatment of kidney disease. The expression of exosomal ECE1 and PDE1A mRNAs, which is downregulated in patients with IgAN who respond poorly to RASi therapy, might serve as biomarkers for predicting RASi therapy efficacy in reducing proteinuria in patients with IgAN [63].

While miRNAs and mRNAs are among the most explored types of uEV content in kidney disease, other RNA species (tsRNAs, circRNAs, and lncRNAs) have also received increasing attention in recent research, highlighting the potential of various RNA species as biomarkers for kidney diseases.

### Proteins in urinary EVs as biomarkers of kidney disease

Various proteomic technologies have been used to identify proteins dysregulated in the uEVs of different patient groups [84–89].

#### Genetic and congenital diseases

Several dysregulated proteins in uEVs have been identified as biomarkers for genetic and congenital diseases. In addition to ADPKD, the most common inherited kidney disease [90], the protein content of uEVs has also been investigated in a rare genetic disorder [84]: autosomal dominant tubulointerstitial kidney disease-subtype hepatocyte nuclear factor 1β (ADTKD-HNF1β) is caused by mutations in the *HNF1β* gene leading to renal and extrarenal manifestations [84]. Through proteomics, five differentially enriched proteins were identified in the uEVs of patients with ADTKD-HNF1β compared with those of patients with CKD. Furthermore, enrichment of pathways related to cilia and cytoskeletal organization, as well as depletion of several members of the serpin superfamily, was found in the uEVs of patients with ADTKD-HNF1β and ADPKD compared with those in patients with CKD. Analysis of the uEV proteome provided new insights into the pathophysiology of ADTKD-HNF1β and revealed that ADTKD-HNF1β resembles ADPKD more closely [84]. Furthermore, vesicular MMP-7 was discovered as a potential biomarker for the identification of rapid disease progressors in ADPKD [90]. Furthermore, Bruschi *et al.* reinterrogated raw proteomic data obtained from urinary EVs from patients with medullary sponge kidney (MSK) disease, a rare congenital malformation, and those with idiopathic calcium nephrolithiasis (ICN). Statistical algorithms have identified different ephrin receptors as the most promising biomarkers to distinguish between patients with MSK and those with ICN [88].

#### Metabolic diseases

In addition to different genetic factors that may lead to CKD, metabolic and systemic factors lead to the development of CKD. Specifically, diabetes and hypertension are the leading causes of CKD in all developed countries and many developing countries [91]. Changes in the proteomic profile of the uEVs of patients suffering from these conditions have been investigated extensively.

Numerous dysregulated proteins were identified in the uEVs of patients with hypertension with an albumin creatinine ratio (ACR) <10 mg/g versus those of patients with hypertension with an ACR of 10–30 mg/g, i.e. in the upper normal range. In a validation cohort, SLC27A2 and amnionless were upregulated in the uEVs of the latter, underscoring their potential as predictive biomarkers of kidney disease before pathological ACR levels are reached [85].

Employing mass spectrometry, Liu *et al.* determined the expression of aerobic oxidative metabolic proteins in uEVs. Among a broad array of identified proteins, GAPDH, PFKM, ACO2, and MDH2 were downregulated in patients with diabetes compared with healthy controls, whereas IDH3G was increased. The downregulated proteins might be used either alone or in combination to monitor changes in diabetes [87]. PAK6 and EGFR, which are significantly more abundant in the uEVs of patients with diabetic nephropathy than in those of healthy controls and patients with T2DM, may be biomarkers for diagnosing diabetic nephropathy [86]. uEV-NGAL, which is present in the EV fraction of children and adolescents with T1DM but is undetectable in healthy controls, may serve as an early biomarker for DKD [92].

Phosphorylation events are critical drivers of the initiation and progression of kidney disease, offering more precise insight into the disease status than total protein quantification does. Accordingly, the presence of phosphoproteins in uEVs may serve as diagnostic and prognostic biomarkers of kidney disease. Numerous differentially expressed uEV phosphoproteins were recently identified between patients with DN and patients with diabetes with normal kidney function, patients with DN and healthy controls and patients with diabetes with normal kidney function and healthy controls. Among the 47 differentially expressed proteins between DN patients and patients with diabetes, phosphorylated aquaporin-2 [p-AQP2(S256)] and phosphorylated glycogen synthase kinase-3β [p-GSK3β(Y216)] were initially verified, suggesting their potential as biomarkers of diabetic nephropathy [93].

#### Glomerular diseases

The expression of vesicular vasorin and ceruloplasmin differed among patients with IgAN, patients with membranous glomerulonephritis, and healthy controls. Furthermore, both EV and podocyte-related proteins were significantly more highly expressed in the uEVs of patients with glomerulonephritis [94]. MASP2 expression was also significantly elevated in the uEVs of patients with IgAN compared with those of healthy controls [60]. Changes in the aforementioned proteins may indicate podocyte/kidney injury [60, 94]. In an interesting approach, Cricri *et al.* investigated the expression of surface markers of EVs in the serum and urine samples of children with INS and healthy controls using flow cytometry [56]. Various uEV surface markers in various combinations or alone have been shown to be able to separate/differentiate among patients with different INS subgroups based on, for example, steroid sensitivity or disease activity. Notably, combining serum and urine EV surface markers results in greater discriminatory power compared with single markers [56].

#### CKD

Osteopontin, which was identified via mass spectrometry, and its N-terminal fragment (N-OPN) were significantly upregulated in the uEVs of patients with CKD. These results lead to the assumption that N-OPN may be an indicator of CKD progression and renal fibrosis [89].

In summary, several promising biomarkers have been identified in the protein cargo of uEVs, which need to be validated in further studies with a greater number of patients. Furthermore, analysis of the proteomic cargo in uEVs may help to gain new insights into the pathophysiology of various kidney diseases.

### Other biomarkers in uEVs

In addition to proteins, other molecules have potential as biomarkers for kidney diseases. For example, the ratio of uEV-cAMP to total urinary cAMP was elevated in ADPKD patients compared with controls. Furthermore, uEV-cAMP showed a positive association with kidney volumes below 1 l per 1 m of height, followed by a drastic decrease in its concentration at larger kidney volumes/larger renal sizes [95].

Other approaches to assess uEVs as markers for different kidney diseases include Raman spectroscopy, which provides information about the chemical composition based on the detection of functional groups. Analysis of the obtained EV-Raman spectra allowed the differentiation of patients with diabetes with different stages of CKD and healthy controls [96].

## CONCLUSION

uEVs have gained significant attention as sources of biomarkers, as their cargo reflects the state of the cells from which they originate. The combination of markers of specific renal cells in uEVs that provide information about altered cellular function and/or viability may reveal the localization, causes, and longitudinal progression of various kidney diseases. Furthermore, investigations of EV subtypes derived from specific cells may lead to the identification of new biomarkers. In addition to the content of uEVs, the size and size distribution of specific uEV subtypes appear to be altered in various kidney diseases and may predict the progression of kidney disease.

Most of the mentioned studies were cross-sectional, thus limiting their potential to identify biomarkers of uEVs with prognostic value. Consequently, future longitudinal studies are needed to explore the development of biomarkers over time and establish new biomarkers to predict the progression of kidney disease. Hypothetically, readily accessible biomarkers in uEVs may be implemented in routine preventive examinations to predict the risk of kidney disease development and, consequently, provide a rationale for the early initiation of renoprotective interventions.

## Data Availability

No new data were generated or analyzed in support of this research.
